# Child Life under Queen Victoria

**Published:** 1897-04-24

**Authors:** 


### Child Life under Queen Victoria.

### XI.—THE SOCIETY FOR THE PREVENTION OF CRUELTY TO CHILDREN.

All our previous articles have shown, in varying circumstances of wrong, one course of remedy—the State interfering between parent and child or between employer and child, to prevent cruelty, injustice, or neglect. In the last resort the State must take upon itself the office of guardian of its growing citizens; but before the State comes the family, and it is not fair, it is not wholesome, that the State should supersede the family, or lay upon the shoulders of the community at large the burdens and responsibilities which men and women voluntarily and naturally take upon themselves when they become parents. The tendency of modern sentiment is too much to call upon the State to do the work of the parent; and it may have appeared that the writer of these articles approved the tendency, for in them it was unavoidable that stress should be laid on the value and necessity of State intervention. Therefore, it has seemed desirable to conclude by an account of a society, the work of which is to make parents do their own work and bear their own burdens. We mean the National Society for the Prevention of Cruelty to Children.

Legislation in general must deal with its objects *en masse*. It defends the factory worker as factory worker, the collier as collier, and so forth. But before the child can become a worker at all and as such claim the law's protection in its work, it is—in something called or mis-called a home—a child. It needs food, clothing, tendance, and, thank God! it gets in many homes, even in the poorest, much more than these; but there are others! It was to deal with these others that this society was formed.

It is at the time of writing only some eight years old, but it has already availed to alter the general notion that what was done in the home was no concern of anyone's outside. The society meant to deal with children not in the mass but as individuals. But the first step to this was to give children a legal status. Prior to 1889 a child could not give evidence against its parents, nor could one parent give evidence against the other. With such a law it is evident that, whilst morally there might be no doubt that a child was neglected and ill-used—while its appearance might bear most eloquent testimony to its sufferings, the most sympathetic Judge had no alternative but to disallow its plea. This Act — the "Children's Charter" as it has been called—revolutionised the condition of children. The child became at its birth a citizen, with a citizen's rights, instead of having to wait till it attained its majority before being able to claim protection from its parents. There were the usual objections of distorted sentimentalism—"an Englishman's house was his castle," even though he kept in his castle a torture-chamber for his babes; the Act would establish a system of domestic espionage; parental discipline would come to an end if every touch of the rod had to be accounted for. Similar objections have been raised to every statute that would ameliorate the condition of one class and control the harshness of another. And it may be admitted that to give one man plenary powers to interfere with the management of another man's family seems, on the face of it, a dangerous experiment. But practice often contradicts theory, and it is impossible to apply the strict rules of logic to human nature.

The action of the Society for the Prevention of Cruelty to Children has never been proved vexatious. It does not interfere with ordinary parental control. But there are cases where all the traditions of parenthood seem to have vanished except the tradition of power; where cruelty and not tenderness mark the relation. In these homes there is usually drunkenness as well, occasionally there is simply wilful laziness; but always the children are suffering. Sometimes, when we find that children's lives are insured—the common phrase implies the reverse of the truth in these cases—there is the absolute intention of killing them; but often enough there is simply the callousness of a debased and selfish nature in father or mother. It is with such cases that the society deals.

The charge of espionage cannot be altogether denied. Secrecy is maintained as to the name of any person who gives information as to cruelty. But, except under this shield of inviolable confidence, how would the kindly but peace-loving neighbour venture on so thankless a task? How would even the terrified mother or the heart-broken father venture to betray the cruelty or neglect of the other parent? But if the society is put on the track by outsiders, it takes action only on what is seen by its own officers. Then the defaulting parent is summoned for cruelty or neglecting to provide for his family, and, if imprisoned, the children are looked after during his absence. After this an official of the society visits the family regularly, warning, reproving, encouraging, as the case may require, and continues his supervision until the erring parent seems to be reformed and the family is well cared for and content.

The strange thing is that this reformation is a real thing. Sometimes, indeed, it requires more than one conviction to prove to the tyrant that cruelty does not pay. But virtue, like vice, becomes easier as it is practised, even though practised only on compulsion. Perhaps it would be more just to say that parental love and duty, though obscured and well-nigh killed by self-indulgence, laziness, or uncontrolled temper, exist in every heart, and can be cultivated even in unpromising soil. Neglect may often spring from mere lack of self-consciousness. Only on this hypothesis can we explain the fact that parents often reform after being merely warned by the society. Ostrich-like, they have shut their eyes to their own sins, and are shocked—often, happily, shocked into better conduct—by realising that it is condemned by others.

The Society for the Prevention of Cruelty to Children claims to have changed the condition of over 50,000 children for the better. Moreover, the effect of even one prosecution as a warning to other neglectful parents is something which can hardly be estimated by statistics, but must be very important. But, perhaps, the most useful part of its work is its determination to call in State aid, not to supersede but to confirm the ties of the family, not to relax but to enforce the bond that ordains that the parent shall provide and care for the child.

